# Ileocecal Mesentery Arteriovenous Malformation as a Rare Cause of Ectopic Variceal Bleeding in a 58-Year-Old Male With Cirrhosis

**DOI:** 10.7759/cureus.45785

**Published:** 2023-09-22

**Authors:** Luis E Santiago, Ali Tariq Alvi, Angelina M Hong, Anthony Pasarin, Pallavi Aneja

**Affiliations:** 1 Internal Medicine, HCA Florida Westside Hospital, Plantation, USA; 2 Internal Medicine, HCA Florida Northwest Hospital, Margate, USA; 3 General Surgery, HCA Florida Westside Hospital, Plantation, USA; 4 General Surgery, HCA Florida Northwest Hospital, Margate, USA

**Keywords:** complications of cirrhosis, alcoholic cirrhosis, diagnosis of ectopic varices, retroperitoneal hematoma, ruptured arteriovenous malformation, ectopic varices

## Abstract

Ectopic varices can be defined as dilated portosystemic venous collaterals that are located at a site other than the esophagus or stomach. These varices can be seen in patients with underlying portal hypertension, but bleeding from them is quite rare. The bleeding usually occurs in patients with a history of intra-abdominal surgery and adhesions. These varices are commonly found in the duodenum or rectum, but they can be present anywhere along the gastrointestinal tract. Currently, there are no well-established guidelines regarding the diagnosis and management of these variceal bleeds, and further investigations with randomized controlled or large-scale trials are required. Here, we report an unusual case of ectopic variceal bleeding from an ileal arteriovenous malformation (AVM), which presented as syncope associated with an acute abdomen in a patient with no prior history of intra-abdominal surgery.

## Introduction

Variceal bleeding involving the esophagogastric region is a frequent complication in patients with portal hypertension. Portosystemic collaterals can form anywhere along the gastrointestinal tract with underlying portal hypertension. Bleeding from these sites is a life-threatening complication for these patients. Collaterals that form within the adhesions can cause ectopic varices, especially in the jejunum and ileum. There are multiple cases of ectopic varices arising from the ileum in the literature, but bleeding from these varices is uncommon, and most of these cases seem to have had prior intra-abdominal surgery with adhesions [1], unlike in this case.

Arteriovenous malformations (AVM) involving the gastrointestinal tract are rare, though they are primarily seen in the small intestine and right hemicolon. The term AVM has been used to report a wide range of vascular lesions of the intestine, including vascular dysplasia, vascular ectasia, telangiectasia, or angiodysplasia [2]. An AVM may instead be seen in patients without prior surgeries, which can unexpectedly cause massive lower gastrointestinal hemorrhage or intraperitoneal hemorrhage [1]. Here, we report an unusual case of ectopic variceal bleeding from an ileocecal mesentery AVM, which presented as syncope associated with an acute abdomen in a patient with no prior history of intra-abdominal surgery.

## Case presentation

We describe a 58-year-old man with a history of gastric ulcers and alcohol abuse who presented to the hospital following a syncopal episode while playing pickleball. He described a sudden onset of diffuse abdominal pain and nausea, followed by a syncopal episode. He denied any hematemesis or melena. On arrival at the emergency department, he was hypotensive, with a blood pressure of 73/34 mmHg. Aggressive intravenous resuscitation was given. On physical examination, there was diffuse abdominal tenderness and guarding on minimal palpation. Initial laboratory investigations revealed hemoglobin of 9.5 g/dl, platelet count of 95 × 109/L, total bilirubin of 5.5 mg/dl, unconjugated bilirubin of 3.2 mg/dl, aspartate aminotransferase (AST) of 82 U/L, and alanine aminotransferase (ALT) of 32 U/L. The coagulation profile showed prothrombin time (PT) of 30.2 seconds, activated partial thromboplastin time (aPTT) of 41.8 seconds, international normalized ratio (INR) of 2.5, and fibrinogen of 87 mg/dl.

A computed tomography (CT) scan of the abdomen and pelvis without contrast performed on arrival showed moderate abdominal free fluid and minor to moderate pelvic free fluid. Computed tomography angiography (CTA) of the abdomen revealed a cirrhotic liver, abdominal varices, a stable amount of abdominal and pelvic free fluid, and no extravasation of contrast upon administration. The abdomen X-ray did not show any evidence of pneumoperitoneum. Therefore, it was decided to manage the patient conservatively for the time being. Due to persistent abdominal pain, gastroenterology and general surgery were consulted to be evaluated further. The next day, hemoglobin dropped significantly to 4.7 g/dl; repeat CTA was performed, which revealed no extravasation of contrast upon administration; however, there were areas of increased free fluid within the abdomen, especially posterior to the inferior right lobe of the liver and within the left paracolic gutter, suggestive of blood products.

**Figure 1:**
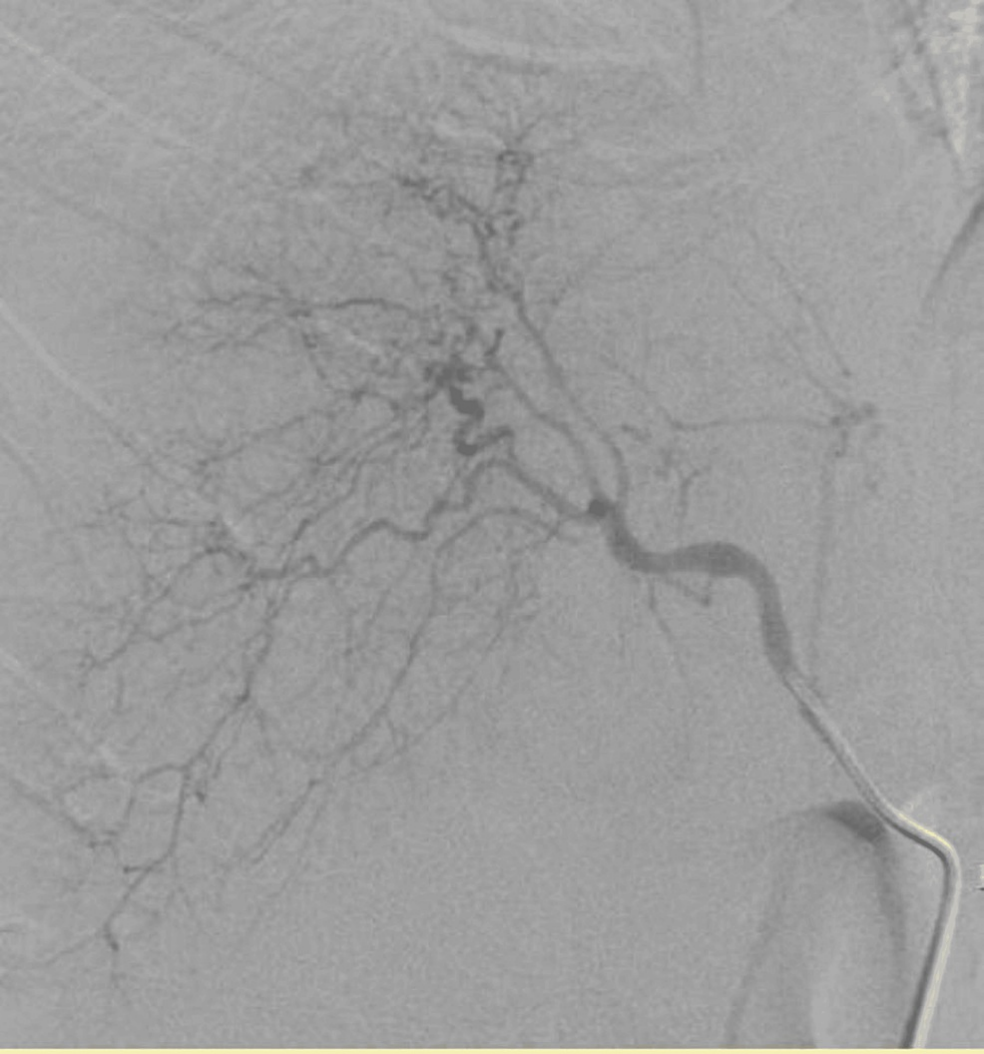
Common hepatic artery angiography. Common hepatic artery angiography showing no evidence of focal blush or extravasation.

**Figure 2:**
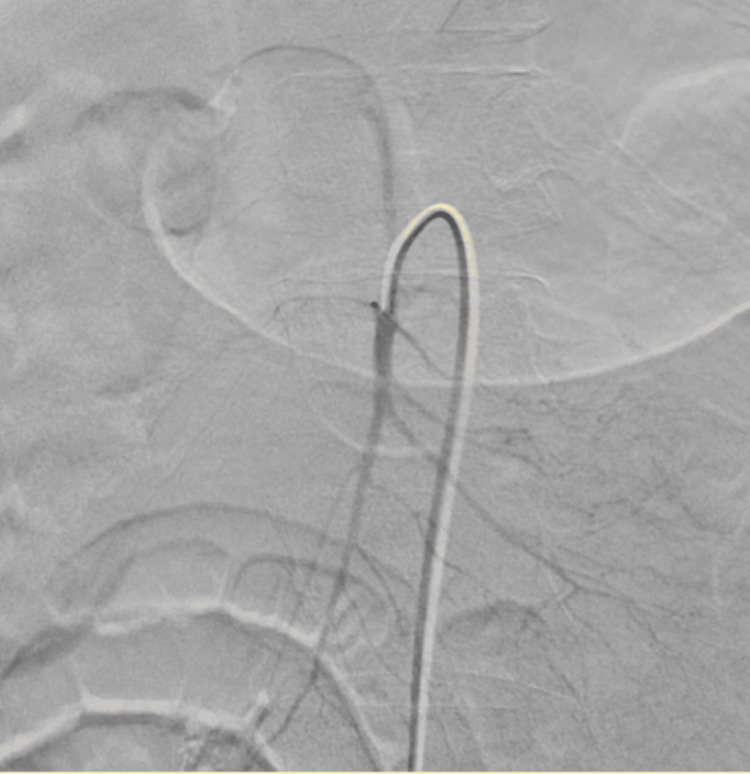
Superior mesenteric artery angiogram. Angiogram of the superior mesenteric artery showing no evidence of focal blush or extravasation.

The patient was transferred to the intensive care unit (ICU) for a higher level of care. With a high suspicion of hepatic bleeding, an angiogram of the celiac artery, superior mesenteric artery (SMA), common hepatic artery, right hepatic artery, and left hepatic artery was performed; the results were unremarkable (Figures 1-2).

**Figure 3:**
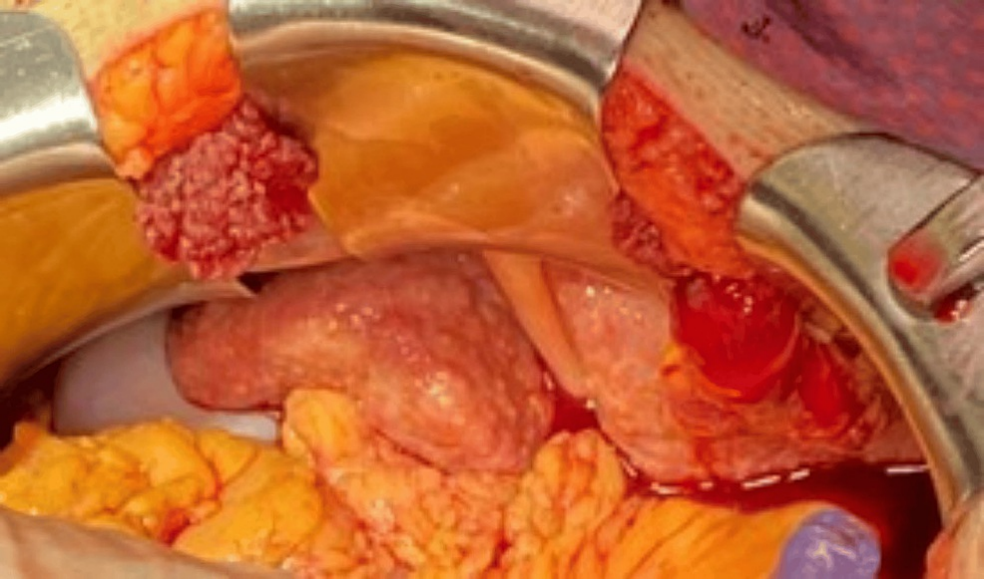
Open abdomen from laparotomy incision. Open abdomen from a laparotomy incision showing a small, cirrhotic, nodular liver in the right upper quadrant of the abdomen and blood.

**Figure 4:**
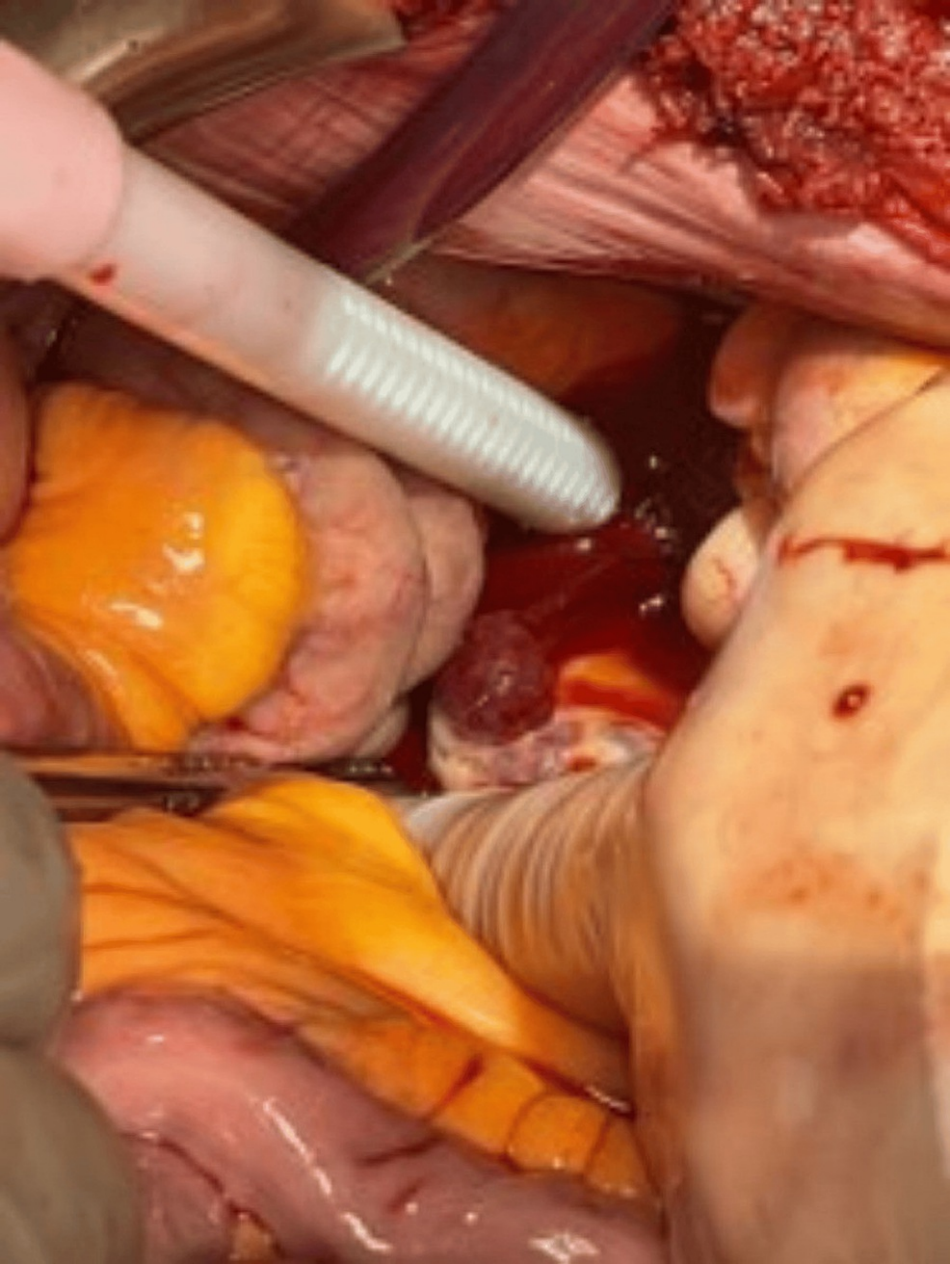
Bleeding from an ileocecal mesentery arteriovenous malformation. Open abdomen from a laparotomy incision showing bleeding from an ileocecal mesentery arteriovenous malformation.

The patient became hemodynamically unstable and was transfused multiple units of packed red blood cells, fresh frozen plasma (FFP), and cryoprecipitate, but his condition continued to deteriorate. Therefore, the patient was taken for an emergent laparotomy. Upon entering the abdomen through a subcostal incision, we noted copious amounts of blood. After removing all clots from the abdomen, we irrigated all four quadrants. The liver was noted to be contracted and cirrhotic with no active bleeding (Figure 3). There was also no bleeding in the left upper and left lower quadrants. Upon evaluation of the right lower quadrant, persistent and copious venous bleeding was noted. After careful inspection, we found a large bleeding AVM within the ileocecal mesentery (Figure 4), which was successfully ligated with a figure of eight sutures. Unfortunately, the patient developed disseminated intravascular coagulation (DIC) due to severe coagulopathy and expired postoperatively.

## Discussion

Ectopic varices are abnormally dilated collateral veins located in unusual sites other than the gastroesophageal region [3]. These collaterals can arise from anywhere in the portal venous system. Typically, the resistance is high in these collaterals compared to the portal venous system, which has low resistance. However, when intrahepatic portal hypertension occurs, the high resistance in the portal system diverts blood toward these collaterals. Approximately 1% to 5% of all ectopic variceal bleeds occur in patients with intrahepatic portal hypertension and 20% to 30% in patients with extrahepatic portal hypertension [4]. These varices are difficult to localize; currently, there are no established guidelines for managing bleeding ectopic varices. There are numerous ways they can manifest clinically. One of the most severe phenotypes of bleeding ectopic varices is the rupture of retroperitoneal varices, leading to hemoperitoneum, with mortality estimated to be around 70% [5]. In these patients with ectopic variceal bleeding, survival is predicted by functional hepatic reserve, the severity of hemorrhagic shock on initial evaluation, and the time elapsed to operative intervention and control of the bleeding [5]. We present a case of massive hemoperitoneum from an AVM in the ileocecal mesentery of a 58-year-old male with cirrhosis.

We performed an extensive workup on this patient, including abdominal CTA, abdominal CT, and a hepatic angiogram, which did not reveal the source of bleeding. Eventually, an exploratory laparotomy revealed the source. Numerous imaging modalities are used to diagnose these varices, but no established guidelines exist. A CTA of the abdomen and pelvis with contrast aids in the diagnosis, but case reports have shown that it may fail to identify the specific bleeding source due to the intermittent nature of these bleeds, as in our case [6]. Minowa et al. summarized 21 published cases of ileal variceal bleeding. According to their report, superior mesenteric vein angiography was used in 61.9% of the reviewed cases to make the diagnosis. However, in recent cases, multi-detector computed tomography (MDCT) or a combination of MDCT and angiography have been used for diagnosis [7]. Capsule endoscopy is another modality that has been found to detect jejunal and small bowel varices successfully but was not performed due to hemodynamic instability [8,9]. Balloon enteroscopy or push enteroscopy is helpful in visualizing and allowing for the intervention of varices in the small bowel [10]. Endoscopic ultrasound has been used to evaluate rectal [11] and biliary varices [12]. Color Doppler ultrasound is helpful in the diagnosis of umbilical, duodenal [13], rectal [14], gall bladder [15], and choledochal varices [16]. Due to its rapid and widespread availability, MDCT with intravenous contrast should be considered the primary diagnostic method for ectopic varices.

Current recommendations for treating these varices are derived from case reports and small retrospective studies. Minowa et al. reported that 76.1% of their reviewed cases of ileal variceal bleeding were treated with surgical resection. Other patients underwent other treatment methods, such as transjugular intrahepatic portosystemic shunt (TIPS) and balloon-occluded retrograde transvenous obliteration (BRTO) [7]. Reports show re-bleeding rates of 23-39% in TIPS and 5-16.6% in BRTO for cases of ectopic gastrointestinal variceal bleeding [17]. While minimally invasive therapeutic procedures may be preferred for ectopic gastrointestinal variceal bleeding in high-risk patients with co-morbidities, surgical resection of the affected intestine is currently a safe and effective treatment strategy to prevent further re-bleeding [17]. In our case, we ligated the ileocecal AVM. We did not choose surgical resection as there were concerns about disseminated intravascular coagulation and hemodynamic instability.

## Conclusions

Ectopic variceal bleeding is an uncommon but important differential to consider in patients with portal hypertension who present with abdominal pain, syncope, and hemodynamic compromise. These varices rarely bleed, but if bleeding develops, it is primarily seen in patients with prior intra-abdominal surgery and adhesions. An AVM may instead be seen in patients without prior surgeries, which can unexpectedly cause massive lower gastrointestinal bleeding. The patient we described in this case report developed ectopic variceal bleeding associated with an ileocecal mesentery AVM with no prior history of intra-abdominal surgery. Through this case, we hope to alert clinicians to consider the presence of ectopic variceal bleeding as one of the possibilities in such circumstances.
